# Induction of wheat straw delignification by *Trametes* species

**DOI:** 10.1038/srep26529

**Published:** 2016-05-24

**Authors:** Aleksandar Knežević, Mirjana Stajić, Vladimir M. Jovanović, Višnja Kovačević, Jasmina Ćilerdžić, Ivan Milovanović, Jelena Vukojević

**Affiliations:** 1 University of Belgrade, Faculty of Biology, Takovska 43, 11000 Belgrade, Serbia; 2 University of Belgrade, Institute for Biological Research “Siniša Stanković” Bulevar Despota Stefana 142, 11060 Belgrade, Serbia

## Abstract

Wheat straw is the major crop residue in European countries which makes it the most promising material for bioconversion into biofuels. However, cellulose and hemicellulose are protected with lignin, so delignification is an inevitable phase in lignocellulose processing. The organisms predominantly responsible for its degradation are white-rot fungi and among them *Trametes* species represent promising degraders due to a well-developed ligninolytic enzyme system. Although numerous studies have confirmed that low molecular weight compounds can induce the production and activity of ligninolytic enzymes it is not clear how this reflects on the extent of delignification. The aim of the study was to assess the capacity of *p*-anisidine and veratryl alcohol to induce the production and activity of Mn-oxidizing peroxidases and laccases, and wheat straw delignification by six *Trametes* species. Significant inter- and intraspecific variations in activity and features of these enzymes were found, as well as differences in the potential of lignocellulose degradation in the presence or absence of inducers. Differences in the catalytic properties of synthesized enzyme isoforms strongly affected lignin degradation. Apart from enhanced lignin degradation, the addition of *p*-anisidine could significantly improve the selectivity of wheat straw ligninolysis, which was especially evident for *T. hirsuta* strains.

Lignocellulose is a major component of plant biomass, composed of cellulose, hemicellulose and lignin, and represents the most abundant renewable organic source in ecosystems^1^,^2^. Huge amounts of this potentially valuable material are produced in agriculture and forestry but only 3% of annual production is used directly while the rest is considered as waste^3^. Wheat straw is the major crop residue in European countries with an annual production of around 170 × 10^6^ tons, which makes it the most promising material for bioconversion into food, feed, chemicals and biofuels^4^. However, cellulose and hemicellulose, as good carbon and energy sources, are protected with lignin, the most recalcitrant polymer that gives mechanical resistance to wheat straw and represents a barrier to access these polymers^1^,^2^. Thus, delignification is an inevitable phase in lignocellulose processing^5^. As chemical and physical decomposition of lignins are neither environmentally friendly nor economically justified processes, biological degradation is becoming an excellent alternative. Although some groups of bacteria can degrade lignin, the organisms predominantly responsible for its mineralization are white-rot fungi^6^,^7^. Among them, species of the genus *Trametes* are promising lignin degraders due to their well-developed ligninolytic enzyme systems comprised of Mn-oxidizing peroxidases and laccases^8^.

Ligninolysis is an extracellular and oxidative process which requires the synthesis of ligninolytic enzymes and lignin depolymerisation followed by degradation of the resultant aliphatic and aromatic components^1^. The production and activity of these enzymes are highly regulated by physical and chemical factors including the presence of inducers, low molecular weight compounds that affect gene transcription or act as chemical mediators^9^,^10^. Previous studies have shown that among various inducers, veratryl alcohol and *p*-anisidine possess the ability to stimulate both laccase and peroxidase activity in white rot fungi^11^,^12^,^13^,^14^,^15^. Thus, the addition of such compounds during fermentation of plant residues can be an excellent approach for accelerating enzymatic reactions during pre-treatment of lignocellulosic wastes. Although numerous studies have confirmed that these compounds can induce the production and activity of ligninolytic enzymes^11^,^13^,^14^,^15^,^16^ it is not clear how this reflects on the extent of lignin degradation.

The aim of this study was to assess the capacity of *p*-anisidine and veratryl alcohol to induce the production and activity of Mn-oxidizing peroxidases and laccases, as well as wheat straw delignification by six *Trametes* species.

## Results and Discussion

### Variation in enzyme activities

**Figure 1:**
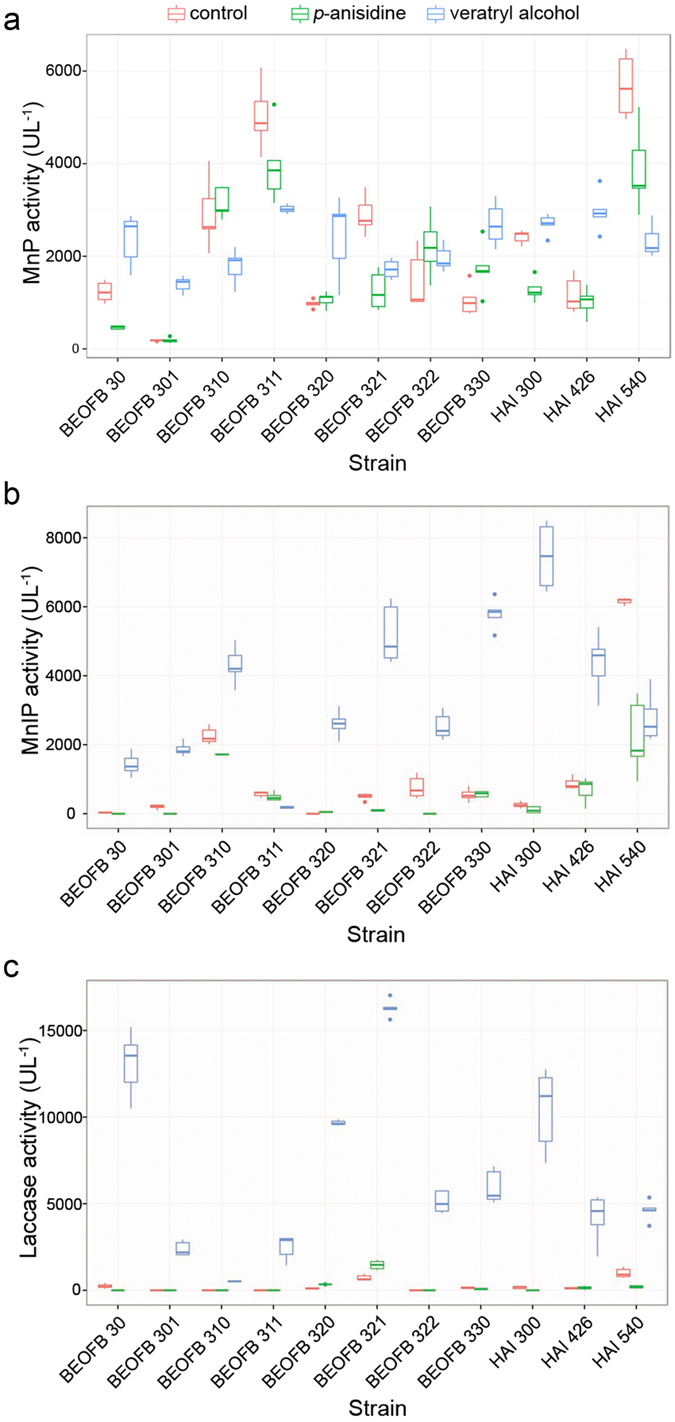
Activity of Mn-dependent peroxidase (**a**), Mn-independent peroxidase (**b**) and laccase (**c**) in *Trametes* spp. strains depending on treatment with inducers. Boxplots represent the median, interquartile range, and minimum to maximum range of enzyme activity level, n = 5.

The six *Trametes* species and 11 strains showed various levels of ligninolytic enzyme activities as a result of treatment with *p*-anisidine and veratryl alcohol (Fig. 1). The highest MnP activity in the absence of inducers was found in *T. multicolor* HAI 540 (5683.8 ± 439.1 U L^−1^) and the lowest in *T. hirsuta* BEOFB 301 (181.8 ± 8.7 U L^−1^) (Supplementary Table S1). Addition of *p*-anisidine to the medium increased MnP activity in *T. gibbosa* BEOFB 310 and *T. versicolor* BEOFB 322. However, in most strains this inducer either negatively affected MnP activity or had no significant effect (Fig. 1a). On the other hand, the presence of veratryl alcohol in the medium increased MnP activities in most strains. A negative effect of this inducer on MnP activity was noted in *T. gibbosa* BEOFB 310 and BEOFB 311, *T. multicolor* HAI 540 and *T. versicolor* BEOFB 321 (Fig. 1a), the strains that in the control had activities over 2000 U L^−1^.

The highest level of MnIP activity, in the absence of inducers, was also found in *T. multicolor* HAI 540 (6160.9 ± 70.7 U L^−1^) (Supplementary Table S1). *p*-anisidine suppressed the activity of this enzyme compared with the control, with the exception of *T. versicolor* BEOFB 320 where a slight increase of activity was detected, and *T. multicolor* HAI 426 and *T. pubescens* BEOFB 330 where there was no significant effect on the enzyme activity. Contrary to this, veratryl alcohol had a stimulatory effect on MnIP activities in all strains except *T. gibbosa* BEOFB 311 and *T. multicolor* HAI 540 (Fig. 1b).

Laccase activity in the absence of inducers was very low in all strains, but in *T. multicolor* HAI 540 it was the highest, as was the case with peroxidases (Fig. 1c). A slight positive effect of *p*-anisidine on the activity was detected only in *T. versicolor* BEOFB 320 and BEOFB 321, and a suppressed effect in *T. multicolor* HAI 540, while no significant variability among other strains was noted. Veratryl alcohol significantly induced the activity of laccase in all strains and especially in *T. versicolor* BEOFB 321 (Fig. 1c), where the increase was highest compared with the control, reaching even 22.7-fold (16291.4 ± 405.9 U L^−1^ *vs.* 716.7 ± 121.1 U L^−1^) (Supplementary Table S1).

Two-way analysis of variance of MnP, MnIP and laccase activities showed that strain, inducer type, as well as their interaction were significant factors which affected the activities of these enzymes. The F statistic for MnP was F_32,132_ = 38.45, P < 0.001; for MnIP was F_32,132_ = 38.45, P < 0.001 and for laccase was F_32,132_ = 38.45, P < 0.001.

**Figure 2:**
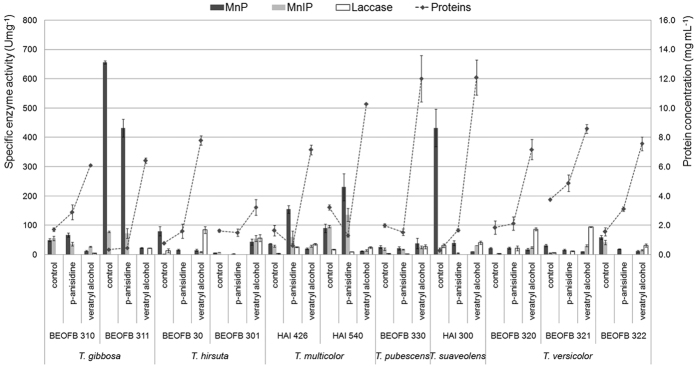
Protein production and specific activity of Mn-oxidizing peroxidases and laccase in *Trametes* spp. strains depending on treatment with inducers. Data represent mean ± S.E., n = 5.

The two inducers significantly affected total protein production which was reflected in specific activities of the enzymes studied (Fig. 2). Total protein production was higher in the presence of veratryl alcohol and the maximum was noted in *T. pubescens* BEOFB 330 and *T. sualveolens* HAI 300 (12.0 ± 1.6 mg mL^−1^) which had a 6-fold higher concentration than under control conditions (2.0 ± 0.1 mg mL^−1^). Under control conditions, the highest specific MnP activity was present in *T. gibbosa* BEOFB 311 (656.6 ± 5.6 U mg^−1^), slightly lower in *T. sualveolens* HAI 300 (432.2 ± 63.5 U mg^−1^), while in all other strains MnP activities were below 100.0 U mg^−1^. The two inducers had no significant effect, except in the case of *p*-anisidine which showed a positive effect in *T. gibbosa* BEOFB 311 (431.9 ± 30.4 U mg^−1^) and *T. multicolor* HAI 540 and HAI 426 (230.6 ± 46.0 U mg^−1^ and 154.4 ± 13.4 U mg^−1^, respectively).

MnIP was not expressed under control conditions in nearly all strains, except *T. multicolor* HAI 540, where the maximum activity was found (95.6 ± 3.9 U mg^−1^), *T. versicolor* BEOFB 322 (41.7 ± 7.5 U mg^−1^) and *T. suaveolens* HAI 300 (19.4 ± 4.2 U mg^−1^). *p*-anisidine stimulated specific MnIP activity only in *T. multicolor* HAI 540 (135.9 ± 21.8 U mg^−1^) which was about 30% higher than in the control, while in all other strains its effect was either absent or inhibitory, as in the cases of *T. versicolor* BEOFB 322 and *T. suaveolens* HAI 300. Veratryl alcohol showed similar effects, and expressed the enzyme only in the case of *T. hirsuta* BEOFB 301, where the specific activity was 60% higher than in the control (54.9 ± 10.3 U mg^−1^) (Fig. 2). In the control and in the presence of *p-*anisidine, specific laccase activity was almost absent, while veratryl alcohol had a stimulatory effect, especially in *T. versicolor* BEOFB 321, where the maximum was reached (95.0 ± 1.4 U mg^−1^), *T. hirsuta* BEOFB 30 and *T. versicolor* BEOFB 320 (about 90 U mg^−1^) (Fig. 2).

Significant inter- and intraspecific variations were found in the activities of Mn-oxidizing peroxidases and laccase within the genus *Trametes* in the presence and the absence of inducers. Significant interspecific differences were primarily present in MnP production. Namely, contrary to *T. multicolor* where this enzyme was the most active, the activity obtained in fermentation of wheat straw/(NH_4_)_2_SO_4_/wheat bran medium with *T. hirsuta* was about 30-fold lower, which is in accordance with results reported by Erden *et al*.^17^ for *T. versicolor* and *T. hirsuta*.

The effect of inducers on the activities of ligninolytic enzymes has been the subject of numerous studies^9^,^10^,^11^,^15^,^16^,^18^. Due to their chemical compositions, wheat straw and wheat bran were found to be excellent substrates for both conversions into fungal biomass and ligninolytic enzymes^8^,^19^,^20^,^21^. Namely, wheat straw is rich in cellulose (33.7–47.5%), hemicellulose (25–32%) and lignin (11–23%), as well as in macro- and microelements necessary for enzyme synthesis, and wheat bran contains lignin, phenolic acids and minerals^22^,^23^,^24^,^25^. However, the absence or very low activities of Mn-oxidizing peroxidases and laccase in some cases can be explained by specific physiological characteristics of each strain and by factors which regulate the expression of peroxidase and laccase genes at the transcription level^26^. Thus, it is known that inducers can act as transcription factors affecting the synthesis and activity of ligninolytic enzymes. Galhaup and Haltrich^11^ and Xavier *et al*.^15^ demonstrated the stimulatory effects of *p*-anisidine on laccase activity in *T. pubescens* and veratryl alcohol in *T. versicolor*. However, these findings were not in accordance with results of Galhaup *et al*.^16^ and Bakkiyaraj *et al*.^21^ who reported their inhibitory effects. Positive effects of veratryl alcohol and other inducers on the synthesis of Mn-oxidizing peroxidases in numerous white-rot species were also demonstrated by Kapich *et al*.^13^ and Hakala *et al*.^14^. According to these authors, this inducer stimulated the production of MnP during wheat straw fermentation with *Phanerochaete chrysosporium* and an asparagine-succinate medium with *Physisporinus rivulosus* regulating the transcription of MnP genes.

### Variations in isoenzyme profiles

**Figure 3:**
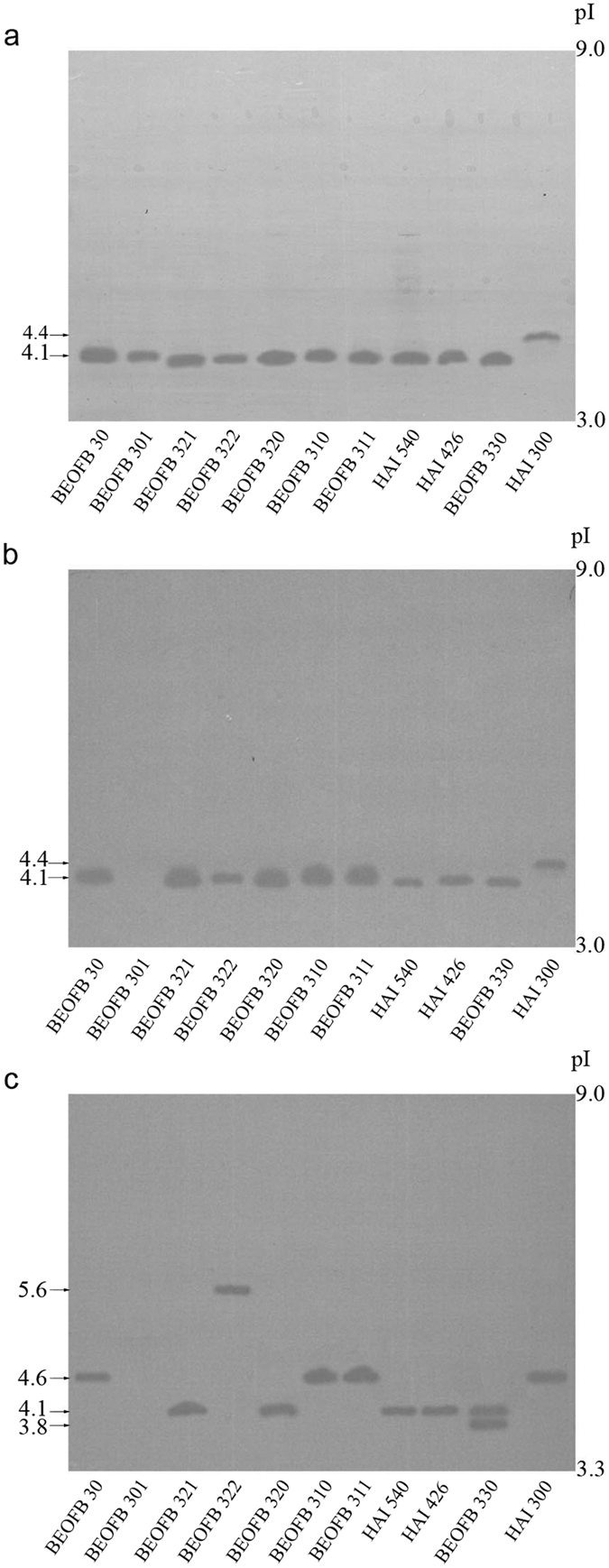
Isoelectric focusing pattern of Mn-dependent peroxidases in *Trametes* spp. strains depending on treatment. (**a**) control; (**b**) *p*-anisidine; (**c**) veratryl alcohol.

After 19-days of solid-state fermentation of wheat straw/(NH_4_)_2_SO_4_/wheat bran medium, strains of *Trametes* species synthesized one MnP isoform of pI around 4.1, except *T. suaveolens* HAI 300 where the pI was 4.4 (Fig. 3a). The presence of *p*-anisidine did not cause any change in MnP isoform profiles except in *T. hirsuta* BEOFB 301, in which it inhibited the synthesis of this enzyme (Fig. 3b). The previously-observed decrease of MnP activity in *T. gibbosa* BEOFB 311, *T. hirsuta* BEOFB 30, *T. multicolour* HAI 540, *T. suaveolens* HAI 300 and *T. versicolor* BEOFB 321 is therefore attributed to decreased enzyme production (Fig. 1a). In contrast, in *T. pubescens* BEOFB 330 there was no difference in enzyme production, while the level of MnP activity increased (Fig. 1a). This, it seems likely that *p*-anisidine acted as a chemical mediator and thus increased the activity of the same MnP isoform. The effect of veratryl alcohol was strain dependent (Fig. 3c). In *T. gibbosa* strains, *T. hirsuta* BEOFB 30, *T. suaveolens* HAI 300 and *T. versicolor* BEOFB 322 this inducer led to the synthesis of MnP isoforms with higher pI values (a maximum pI of around 5.6 was obtained in BEOFB 322), in *T. pubescens* BEOFB 330 veratryl alcohol stimulated the production of an additional isoform (pI 3.8) and completely inhibited the enzyme in *T. hirsuta* BEOFB 301. The induced isoform of pI 4.6 in *T. hirsuta* BEOFB 30 and *T. suaveolens* HAI 300 showed increased levels of enzyme activity, while at the same time less of it was produced (Fig. 1a), which implies that isoforms differ in their catalytic properties. The synthesis of an additional isoform in *T. pubescens* BEOFB 330 was followed by an increase in MnP activity. However, the role of veratryl alcohol as a chemical mediator cannot be ruled out.

**Figure 4:**
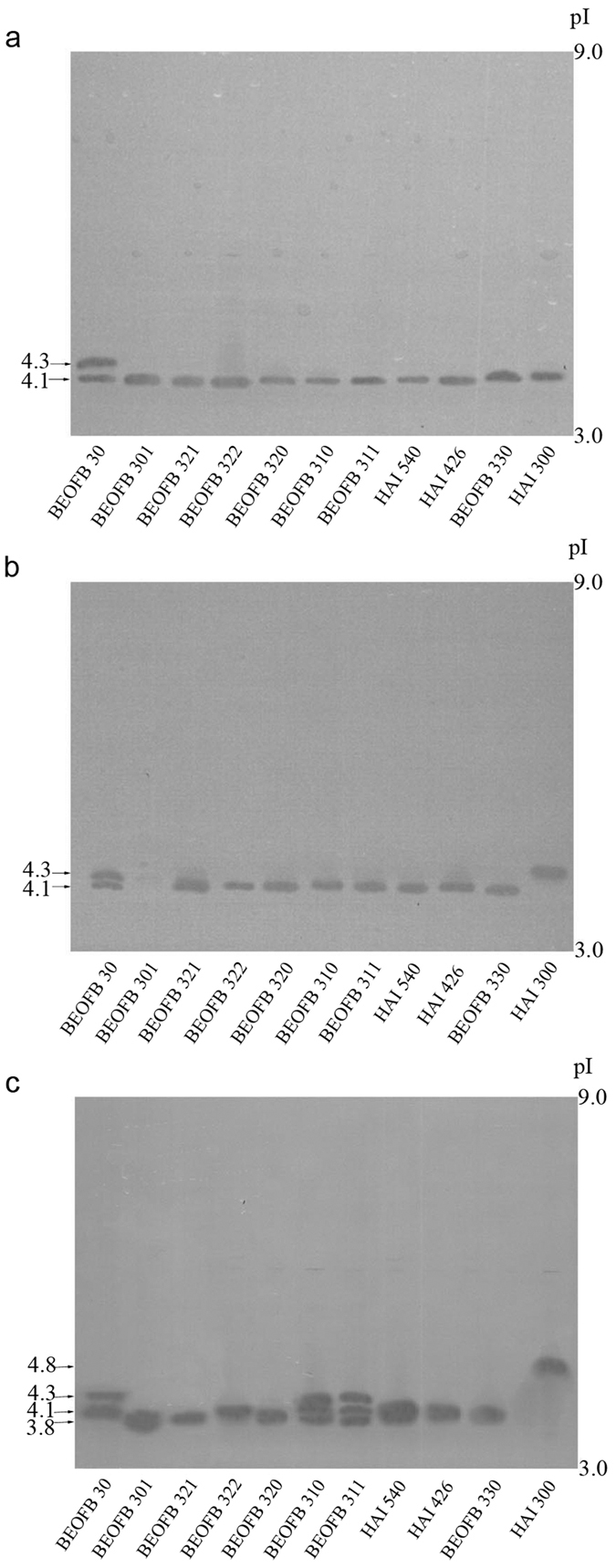
Isoelectric focusing pattern of Mn-independent peroxidases in *Trametes* spp. strains depending on treatment. (**a**) control; (**b**) *p*-anisidine; (**c**) veratryl alcohol.

MnIP production in the *Trametes* spp. strains reacted similarly to the presence or absence of the two inducers (Fig. 4). Thus, all strains synthesized one MnIP isoform of pI 4.1 except *T. hirsuta* BEOFB 30 where one additional isoform of pI 4.3 was detected (Fig. 4a). In the case of *p*-anisidine, production of a pI 4.3 isoform was induced in *T. suaveolens* HAI 300 and enzyme synthesis was inhibited in *T. hirsuta* BEOFB 301 (Fig. 4b). The decrease of MnIP activity in strain HAI 300 could be explained by the lower catalytic properties of the newly synthesized isoform (pI 4.3), despite total protein production being increased in the presence of *p*-anisidine (Figs 1b and 2). Veratryl alcohol stimulated the synthesis of two additional MnIP isoforms in *T. gibbosa* strains (pI around 3.8 and 4.3), caused the production of an isoform with the highest pI in *T. suaveolens* HAI 300 (pI 4.8), and an isoform with a lower pI in *T. hirsuta* BEOFB 301 (pI around 3.8) (Fig. 4c). The increase of MnIP activity noted in the strains that switched isoform production in the presence of veratryl alcohol can be explained not only by increased enzyme production, but also by higher activities of the newly-synthesised isoforms (Figs 1b and 2).

**Figure 5:**
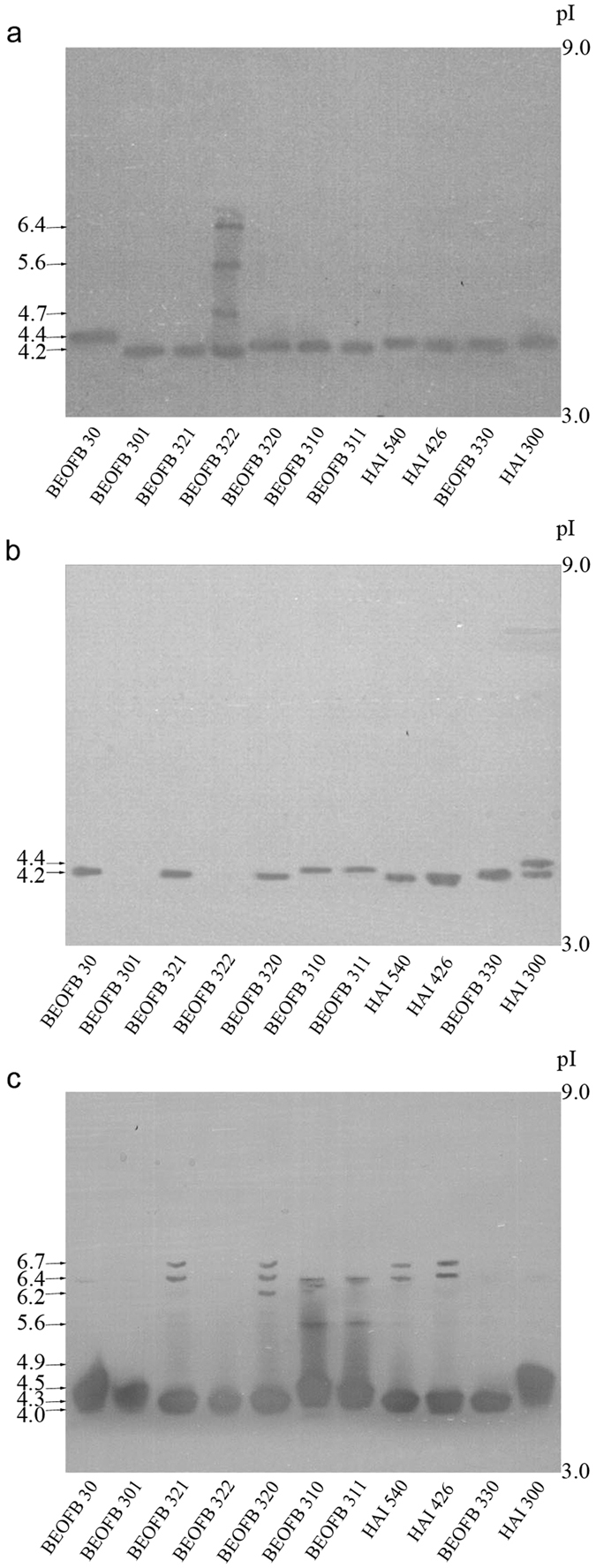
Isoelectric focusing pattern of laccases in *Trametes* spp. strains depending on treatment. (**a**) control; (**b**) *p*-anisidine; (**c**) veratryl alcohol.

Under control conditions, the picture obtained for laccases was more complex (Fig. 5a). Namely, one isoform of pI 4.2 was detected in all strains except in *T. hirsuta* BEOFB 30, which produced an isoform with a pI of 4.4, and in *T. versicolor* BEOFB 322 which synthesized three additional isoforms (pI 4.7; 5.6; 6.3). *p*-anisidine completely inhibited synthesis of the enzyme in *T. hirsuta* BEOFB 301 and *T. versicolor* BEOFB 322, while in *T. suaveolens* HAI 300 production of an additional isoform (pI 4.4) was stimulated (Fig. 5b). However, the effects of veratryl alcohol on laccase synthesis in the strains of *Trametes* species were rather different (Fig. 5c). In most strains, 3–5 new laccase isoforms were detected though some of them were observed as wide bands. Both strains of *T. gibbosa* and *T. multicolor*, as well as *T. versicolor* BEOFB 321 synthesized laccase isoforms of high pI values (6.4 and 6.7) in the presence of the inducer. The appearance of wide bands is likely to be the result of point changes in amino acid sequence or some post-translational modifications of laccase molecules. This effect is expected because laccase synthesis is controlled by families of closely-located genes originating from gene duplication^27^,^28^,^29^. Thus, isoforms with pI 6.4 and 6.7 indicate higher interspecific similarity between *T. multicolor* and *T. versicolor* BEOFB 321. As there were no functional changes, a point mutation could have been the cause of small differences in pI values of laccases encoded by gene families, and this would be reflected through the densely-packed protein bands on the gel (Fig. 5c). In this way, the coloured product of ABTS oxidation could easily have diffused subsequently to make the wide bands. This effect of a single point mutation on the isoelectric point has been theoretically and experimentally shown before for other proteins^30^,^31^.

Although laccase activities were not found spectrophotometrically in *T. gibbosa* strains, *T. hirsuta* BEOFB 301 and *T. versicolor* BEOFB 322, the enzyme was detected by isoelectric focusing in all strains cultivated in the absence of inducers and in the presence of veratryl alcohol, which could be explained either by trace amounts of synthesized enzyme or alternatively by low enzymatic activity of the isoforms produced.

The number of laccase isoforms was best-fitted with two factors in the GLM: treatment and strain (P values were <0.001). Tukey’s multiple post hoc tests showed a significant difference between veratryl alcohol treatment and the control (P < 0.001), and no difference between *p*-anisidine treatment and the control (P = 0.987). Only *T. versicolor* BEOFB 322 showed a significant difference, which was the same with all other strains (P < 0.01).

The level of enzymatic activity was not significantly correlated with all isoforms. The isoform present in the control group (base expression), pI 4.0–4.3, was the one with the weakest effect on the laccase activity detected (Spearman’s correlation coefficient ρ = −0.018, P = 0.02). On the other hand, isoforms synthesized in the treatment with veratryl alcohol (pI 4.3–4.5 and pI 6.7) strongly and positively affected the enzyme activity (P < 10^−10^).

### Variation in capacity of wheat straw depolymerisation

**Figure 6:**
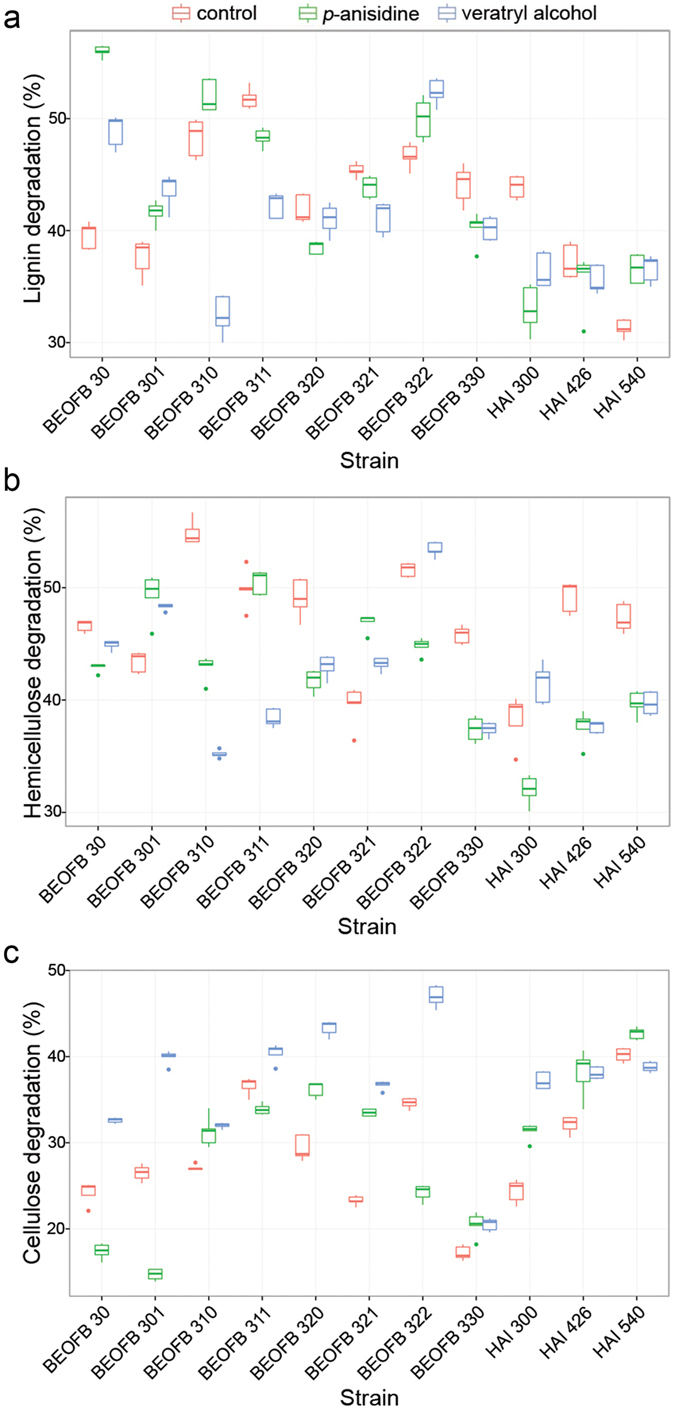
Capacity of lignin (**a**), hemicellulose (**b**) and cellulose (**c**) degradation depending on *Trametes* spp. strains and treatment with inducers. Boxplots represent the median, interquartile range, and minimum to maximum range of enzyme activity level, n = 5.

Proportions of lignin, hemicellulose and cellulose in the wheat straw were 9.7%, 29.5% and 35.5%, respectively. Significant variation in the degradation of these polymers was found and rates of their degradation differed among species and strains and in some cases they were not correlated with the levels of enzyme activities (Fig. 6).

*T. gibbosa* BEOFB 311 had the highest and *T. multicolor* HAI 540 the lowest capacity to degrade lignin under control conditions (51.8% and 31.3%, respectively) (Supplementary Table S2). The addition of inducers to the medium stimulated or inhibited lignin degradation depending on species and strain. *p*-anisidine increased the rate of lignin degradation (from 3.3% to 16.4%) in *T. gibbosa* BEOFB 310, *T. hirsuta* BEOFB 30 and BEOFB 301, *T. multicolor* HAI 540 and *T. versicolor* BEOFB 322, decreased it in *T. gibbosa* BEOFB 311, *T. pubescens* BEOFB 330 and *T. suaveolens* HAI 300, while there was no effect of this inducer on *T. multicolor* HAI 426 and *T. versicolor* BEOFB 321 (Fig. 6a). In the presence of veratryl alcohol, the delignification level increased in *T. hirsuta* BEOFB 30 and BEOFB 301, *T. multicolor* HAI 540 and *T. versicolor* BEOFB 322 (from 5.3% to 9.3%), or decreased in other strains (ranging from 4.2% to 15.9%), having no effect on *T. multicolor* HAI 426 and *T. versicolor* BEOFB 320 (Fig. 6a).

Under control conditions, *T. gibbosa* BEOFB 310 had the highest hemicellulose degradation (54.9%) and *T. suaveolens* HAI 300 the lowest (38.3%) (Supplementary Table S2). Compared with the control, *p*-anisidine increased hemicellulose degradation only in *T. versicolor* BEOFB 321 (7.4%), *T. hirsuta* BEOFB 301 (5.9%), with no significant effect in *T. gibbosa* BEOFB 311 (0.6%), while all other strains were inhibited. Veratryl alcohol increased the amount of degraded hemicellulose from 1.8% to 4.9% in *T. hirsuta* BEOFB 301, *T. versicolor* BEOFB 321 and BEOFB 322 and *T. suaveolens* HAI 300, while in other strains degradation of this polymer was suppressed (Fig. 6b).

Capacities of all the species and strains to degrade cellulose were significantly lower in comparison with their capacities for hemicellulose and lignin depolymerisation. Under control conditions, the highest extent of cellulose degradation was noted in substrate fermentation with *T. multicolor* HAI 540 (40.2%) and lowest with *T. pubescens* BEOFB 330 (17.2%) (Supplementary Table S2). *p*-anisidine in the medium caused the weak induction of cellulose degradation in *T. gibbosa* BEOFB 310, *T. multicolor* HAI 426, *T. pubescens* BEOFB 330, *T. suaveolens* HAI 300, *T. versicolor* BEOFB 320 and BEOFB 321 and decreased degradation in *T. gibbosa* BEOFB 311, *T. hirsuta* stains, *T. versicolor* BEOFB 322 and *T. multicolor* HAI 540. However, the addition of veratryl alcohol to the medium increased the extent of degradation in all species and strains and the amount of degraded cellulose was higher by 2.5% (*T. multicolor* HAI 540) to 18.9% (*T. pubescens* BEOFB 330) (Fig. 6c). *T. multicolor* HAI 426 was the best degrader of cellulose (48.7%, in the presence of veratryl alcohol) and *T. hirsuta* BEOFB 301 the weakest one (14.7%, in the presence of *p*-anisidine).

Two-way analysis of variance of the lignin, hemicellulose and cellulose degradations demonstrated that the strain, inducer and their interaction were statistically significant factors which affected depolymerisation. The overall F statistic for lignin degradation was F_32,132_ = 105.3, P < 0.001, for hemicellulose F_32,132_ = 118.9, P < 0.001, and for cellulose F_32,132_ = 329.7, P < 0.001. Tukey’s post-hoc test revealed significant differences (P < 0.05) between strains in all treatments for each polymer degradation, except for the pair veratryl alcohol – *p*-anisidine in hemicellulose analysis.

Previous studies showed that the amount of degraded lignin during wheat straw fermentation with *T. versicolor* varied between 12.0% after 16 d of cultivation and 42.0% after 35 d^32^,^32^, which was significantly lower in comparison with our data. Lignin degradation was mainly attributed to Mn-oxidizing peroxidases as laccase synthesis was either absent or weakly induced by veratryl alcohol. Mn-oxidizing peroxidases have the main role during the initial phase of delignification as they generate Mn^3+^, while laccase has limited diffusion into the non-modified plant cell wall due to its molecule size. However, in numerous studies of *Trametes* spp. strains, the extent of delignification was not correlated with the level of enzyme activity. Thus, a high capacity of lignin degradation in the presence of *p*-anisidine characterised *T. hirsuta* BEOFB 30, where a low MnP activity and the absence of MnIP and laccase were noted, while *T. multicolor* BEOFB 540 was the weakest lignin degrader both under control conditions and in the presence of inducers, though activities of the enzymes were high. The significant degradation of hemicellulose (32.0–54.9%) and cellulose (14.7–48.7%) is an expected result of substrate fermentation with these white-rot fungal species and strains as they simultaneously degrade lignin and the other two polymers^34^.

The best-fitted minimal GLM for the efficiency of ligninolysis by these six *Trametes* species identified five main factors (strain, type of treatment, and individual activities of MnP, MnIP and laccase) and two significant two-way interactions among them (Table 1). As our results shown, strains reacted with considerable variation to the inducers, so the resulting significance of strain, treatment and their interaction was not surprising.

Looking overall at the efficiency of ligninolysis, *T. pubescens* BEOFB 330 was the most selective lignin degrader under control conditions (rates of degraded lignin and cellulose were 44.1% *vs.* 17.2%). However, in the presence of *p*-anisidine, both *T. hirsuta* strains were the most selective in the sense that cellulose degradation was lower than 20% (rates of degraded lignin and cellulose were 56.0% *vs.* 17.4%, and 41.6% *vs.* 14.7%, respectively).

## Conclusions

Our results clearly showed significant inter- and intraspecific variations in the activities and characteristics of Mn-oxidizing peroxidases and laccases, as well as the potential of lignocellulose degradation within the genus *Trametes* in the presence or absence of inducers. *p*-anizidine and veratryl alcohol can be considered as efficient inducers of these ligninolytic enzymes in *Trametes* species. Differences in the catalytic properties of enzyme isoforms is a factor which strongly affects lignin degradation and this study indicated the most active isoforms. Apart from enhanced lignin degradation, the addition of *p*-anisidine can significantly improve the selectivity of wheat straw ligninolysis which was especially demonstrated for *T. hirsuta* strains. This study demonstrates not only a method to optimize the system for the degradation of wheat straw but also clarifies the role of individual enzymes in lignin degradation by different *Trametes* species and strains.

## Methods

### Organisms and cultivation conditions

Cultures of six *Trametes* species with 11 strains were isolated from fruiting bodies collected from Serbia and Russia or obtained from the culture collection of the Institute of Evolution, University of Haifa, Israel (HAI), and maintained on malt agar medium in the culture collection of the Institute of Botany, Faculty of Biology, University of Belgrade (BEOFB) (Table 2).

The inoculum was prepared by inoculating 100.0 mL of synthetic medium (glucose, 10.0 g L^−1^; NH_4_NO_3_, 2.0 g L^−1^; K_2_HPO_4_, 1.0 g L^−1^; NaH_2_PO_4_ × H_2_O, 0.4 g L^−1^; MgSO_4_ × 7H_2_O, 0.5 g L^−1^; yeast extract, 2.0 g L^−1^; pH 6.5) with 25 mycelial discs (Ø 0.5 cm, from 7-day old culture from malt agar medium), incubation at room temperature (22 ± 2 °C) on a rotary shaker (100 rpm) for 7 d, washing of the obtained biomass with sterile distilled water (dH_2_O), and its homogenization with 100.0 mL of dH_2_O in a laboratory blender.

Solid-state cultivation was carried out at 25 °C in 100-mL flasks containing 1.8 g of wheat straw as the carbon source, 0.2 g of wheat bran as an organic nitrogen source and 10.0 mL of the modified synthetic medium (without glucose and with (NH_4_)_2_SO_4_ at a previously-defined optimum nitrogen concentration of 10.0 mM). The medium with dH_2_O instead of the modified synthetic medium was used as the control. The obtained inoculum (3.0 mL per flask) was used for inoculation. *p*-anisidine or veratryl alcohol were added to the medium 72 h after inoculation to final concentrations of 1.0 mM and 0.5%, respectively, to test their potentials to induce ligninolytic enzyme activity and enhance delignification of wheat straw.

Samples were harvested after 19 d of cultivation and further used for ligninolytic enzyme extraction, while the residues were used for determination of hemicellulose, cellulose and lignin contents.

### Enzyme activity assay and determination of total protein content

Ligninolytic enzymes synthesised during incubation were extracted after solid-state fermentation of wheat straw/wheat bran medium by stirring samples with 50.0 mL of dH_2_O on a magnetic stirrer at 4 °C for 10 min. The extracts were centrifuged (4 °C, 3000 rpm, 15 min) and the supernatants obtained were used for determining Mn-oxidizing peroxidases and laccases activities spectrophotometrically (CECIL CE2501 (BioQuest)), as well as total protein content.

Activities of Mn-oxidizing peroxidases (Mn-dependent peroxidase [EC 1.11.1.13; MnP] and Mn-independent peroxidase [EC 1.11.1.6; MnIP]) were determined with 3.0 mM phenol red (ε_610_ = 22,000 M^−1^ cm^−1^) as a substrate, in a succinate buffer pH 4.5 (succinic acid disodium salt, albumin from bovine serum (BSA) and DL-lactic acid sodium salt). The reaction mixture (V_tot_ = 1.0 mL) contained buffer, sample, 2.0 mM H_2_O_2_ and phenol red, with or without 2.0 mM MnSO_4_ (for MnP and MnIP, respectively). The reaction was stopped by adding 2.0 M NaOH. The reaction mixture using 2.0 M NaOH added before phenol red was used as the blank.

Activity of laccase (EC 1.10.3.2) was determined by monitoring the A_436_ change related to the rate of oxidation of 50.0 mM 2,2′-azino-bis-[3-ethylthiazoline-6-sulfonate] (ABTS) (ε_436_ = 29300 M^−1^ cm^−1^) in 0.1 M phosphate buffer (pH 6.0) at 35 °C. The reaction mixture (V_tot_ = 1.0 mL) contained buffer, ABTS and sample. The mixture without sample was used as the blank.

Enzymatic activity is expressed in U and 1 U is defined as the amount of enzyme that transforms 1.0 μmol of substrate per min.

The amount of total proteins was determined according to the method of Bradford *et al*.^35^. The reaction mixture contained 5.0 mL of Bradford’s reagent and 100.0 μL of sample and absorbance was measured after 5 min of incubation at room temperature at 595 nm. The protein content, presented as mg mL^−1^, was used for determining specific enzymatic activity (U mg^−1^).

### Electrophoresis

The profiles of Mn-oxidizing peroxidases and laccases of the six *Trametes* species and strains were determined by isoelectric focusing (IEF) using the Mini IEF Cell-Model 111 (BIO-RAD). Focusing was carried out in 7.5% polyacrylamide gel with 5.0% ampholyte on a pH gradient from 3.0 to 10.0, in three phases: (*i*) at 100 V, for 15 min; (*ii*) at 200 V, for 15 min; (*iii*) at 450 V, for 60 min. An IEF marker for the pI range from 3.6 to 9.3 (Sigma-Aldrich) was used. Mn-oxidizing peroxidase bands were located by incubating the gel in a solution of 4-Cl-1-naphthol (0.1 mg mL^−1^), 0.05 mM H_2_O_2_ and 0.1 M sodium acetate buffer (pH 4.5), with or without MnSO_4_ (for MnP and MnIP, respectively), at room temperature till the appearance of dark-brown bands. Protein bands corresponding to laccases were located by gel incubation in visualization solution (10.0 mM ABTS and 200.0 mM phosphate buffer pH 5.0) at room temperature. After focusing, the gel was fixed in 12.0% trichloroacetic acid (TCA) and stained with a solution of 0.1% Coomassie brilliant blue R-250 in fixative (methanol, acetic acid and H_2_O in the ratio 45:10:45 v/v/v).

### Determination of hemicellulose, cellulose and lignin content in wheat straw

#### Determination of hemicellulose content

Content of hemicellulose was determined by a modified Van Soest method^36^,^37^. The mixture of dried and ground solid residue (1.0 g), solution of neutral detergent (NDS) (EDTA, 18.6 g L^−1^; SDS, 30.0 g L^−1^; 2-ethoxyethanol, 10.0 mL; NaH_2_PO_4_ × H_2_O, 4.56 g L^−1^; Na_2_B_4_O_7_ × 10H_2_O, 6.81 g L^−1^; pH 6.9–7.1), 0.5 g Na_2_SO_3_ and a few drops of 1-octanol was heated to boiling and then refluxed for an hour to remove soluble sugars, proteins, pectin, lipids and vitamins from the sample. The sample was filtered, washed three times with boiled water and twice with cold acetone, dried at 105 °C for 8 h and weighed as neutral detergent fibres (NDF). The samples were further treated with acidic detergent solution (ADS) (CTAB (20.0 g) dissolved in 1000.0 mL of 0.5 M H_2_SO_4_; pH 6.9–7.1), heated to boiling, refluxed for an hour, filtered and washed with boiled water and a few times with acetone. Thereafter samples were dried at 105 °C overnight and weighed. Weight of acidic detergent fibres (ADF) was determined gravimetrically as the residue remaining after extraction. The hemicellulose content was expressed as ADF-NDF.

#### Determination of cellulose and lignin contents

Acidic detergent fibres were used for determining cellulose and lignin contents. Lignin content was defined by the Klason or 72% H_2_SO_4_ method^38^. 1.0 mL of 72.0% H_2_SO_4_ was added for each 100.0 mg of sample. The mixture was incubated at 30 ± 0.5 °C in a water bath for an hour, stirred frequently and after that diluted using 28.0 mL of water for each 1.0 mL of acid. Secondary hydrolysis was performed in an autoclave at 120 °C for 1 h. The hot solution was filtered through a tared Gooch crucible and the Klason lignin residues were washed with hot water. The sample was dried at 105 °C till constant weight and lignin content (LC) is expressed as a percentage of that present in the original sample. Cellulose content is presented as ADF-LC.

### Statistical analysis

The assays were carried out in five replicates for each measurement occasion and results correspond to the mean ± standard error. Two-way analysis of variance (ANOVA) and Tukey’s HSD post-hoc test were performed to test the significance of differences among mean values of enzyme activities and polymer degradation rates.

Numerical variation in the isoenzyme profiles, as well as in the efficiency of lignin degradation were analysed by generalized linear models (GLM). The difference between percentage of degraded lignin and hydrolyzed cellulose was taken as a measure of the efficiency of ligninolysis for all strains. The full GLM model for isoenzyme profile contained strain, treatment and their interaction as exploratory variables, while full GLM model for efficiency of ligninolysis contained strain, treatment, and activity of ligninolytic enzymes, as well as interactions among these factors. The minimal best fitting model was determined by backward selection, as the model with the lowest Akaike information criterion (AIC). All statistical analyses were performed in R software, ver. 3.2.1.^39^.

## Additional Information

**How to cite this article**: Knežević, A. *et al*. Induction of wheat straw delignification by *Trametes* species. *Sci. Rep.* **6**, 26529; doi: 10.1038/srep26529 (2016).

## Supplementary Material

Supplementary Information

## Figures and Tables

**Table 1:** The best fitting minimal model of significant factors for the efficiency of ligninolysis by studied *Trametes* species.

Model terms	df	Deviance	Residual df	Residual deviance	P^a^
Null model			164	20971.2	
Strain	10	11580.4	154	9390.8	<0.001
Inducer treatment	2	3531.3	152	5859.5	<0.001
MnP activity	1	26.2	151	5833.3	<0.001
MnIP activity	1	152.7	150	5680.6	<0.001
Laccase activity	1	22.8	149	5657.8	<0.001
Strain × Treatment	20	5552.5	129	105.3	<0.001
Strain × MnP activity	10	16.3	119	89.0	0.016

^a^Pearson’s χ^2^ -test.

**Table 2:** Studied *Trametes* species and strains.

Species	Code of strain	Origin of strain
*Trametes gibbosa* (Pers.) Fr.	BEOFB 310	Suva Mt., Serbia
	BEOFB 311	Avala Mt., Serbia,
*Trametes hirsuta* (Wulf.:Fr.) Pil.	BEOFB 30	Novi Beograd, Serbia,
	BEOFB 301	Suva Mt., Serbia
*Trametes multicolor* (Schaeff.) Jülich	HAI 426	Mount Carmel National Park, Israel
	HAI 540	KW, S. Reshetnikov (1570), 1999. Coll. Morkovzi, Kiev, Ukraine
*Trametes pubescens* (Schum.:Fr.) Pil.	BEOFB 330	Verkhnyaya Kvazhva Russia
*Trametes suaveolens* (L.) Fr.	HAI 300	KW, A.S. Buchalo (197), 12/2000. Coll. Belarus
*Trametes versicolor* (L.:Fr.) Lloyd	BEOFB 320	Iverak brdo, Loznica, Serbia
	BEOFB 321	Suva Mt., Serbia
	BEOFB 322	Avala Mt., Serbia
