# Supplementary material for: Induction of wheat straw delignification by Trametes species
Source: Sci Rep. 2016 May 24;6:26529. doi: 10.1038/srep26529 (PMC4877921; doi:10.1038/srep26529)
Supplement: Supplementary Information [file srep26529-s1.doc]

**Induction of wheat straw delignification by *Trametes* species**

Aleksandar Knežević^1*^, Mirjana Stajić^1^, Vladimir M. Jovanović^2^, Višnja Kovačević^1^, Jasmina Ćilerdžić^1^, Ivan Milovanović^1^, Jelena Vukojević^1^

^1^*University of Belgrade, Faculty of Biology, Takovska 43, 11000 Belgrade, Serbia*

^2^*University of Belgrade, Institute for Biological Research “Siniša Stanković” Bulevar Despota Stefana 142, 11060 Belgrade, Serbia*

*Corresponding author:

University of Belgrade, Faculty of Biology, Takovska 43, 11000 Belgrade, Serbia

Tel: +381 11 3244 847; Fax: +381 11 3243 603

e-mail: [knezevica@bio.bg.ac.rs](mailto:knezevica@bio.bg.ac.rs)

**Supplementary file**

**Supplementary Table S1.** Activity of Mn-dependent peroksidase, Mn-independent peroxidase and laccase in tested *Trametes* spp. strains depending on treatment with inducers. Data represent mean ± S.E., n=5.

| Species | Strain | Cultivation conditions | Enzyme activity [UL^-1^] | | |
| --- | --- | --- | --- | --- | --- |
|  |  |  | MnP | MnIP | laccase |
| *Trametes gibbosa* | BEOFB 310 | control | 2917.6 ± 591.7 | 2260.6 ± 171.0 | 0.0 ± 0.0 |
|  |  | *p*-anisidine | 3148.6 ± 166.7 | 1726.7 ± 13.9 | 0.0 ± 0.0 |
|  |  | veratryl alcohol | 1781.6 ± 286.5 | 4302.6 ± 586.7 | 512.0 ± 13.9 |
|  | BEOFB 311 | control | 5030.6 ± 561.4 | 573.7 ± 54.6 | 0.0 ± 0.0 |
|  |  | *p*-anisidine | 3960.9 ± 663.6 | 491.3 ± 30.1 | 0.0 ± 0.0 |
|  |  | veratryl alcohol | 3021.8 ± 65.8 | 186.3 ± 33.4 | 2485.8 ± 517.7 |
| *Trametes hirsuta* | BEOFB 30 | control | 1232.6 ± 208.7 | 34.1 ± 9.3 | 238.9 ± 100.0 |
|  |  | *p*-anisidine | 460.1 ± 27.8 | 0.0 ± 0.0 | 0.0 ± 0.0 |
|  |  | veratryl alcohol | 2368.6 ± 394.3 | 1429.5 ± 241.3 | 13083.2 ± 1369.8 |
|  | BEOFB 301 | control | 181.8 ± 8.7 | 198.8 ± 51.1 | 0.0 ± 0.0 |
|  |  | *p*-anisidine | 183.7 ± 45.2 | 0.0 ± 0.0 | 0.0 ± 0.0 |
|  |  | veratryl alcohol | 1393.5 ± 126.0 | 1872.5 ± 151.2 | 2389.1 ± 292.6 |
| *Trametes multicolor* | HAI 426 | control | 1172.0 ± 268.1 | 874.7 ± 134.0 | 130.8 ± 28.4 |
|  |  | *p*-anisidine | 1009.1 ± 233.0 | 698.6 ± 134.5 | 142.2 ± 79.0 |
|  |  | veratryl alcohol | 2966.9 ± 350.8 | 4379.3 ± 662.6 | 4180.9 ± 320.5 |
|  | HAI 540 | control | 5683.8 ± 439.1 | 6160.9 ± 70.7 | 995.5 ± 183.1 |
|  |  | *p*-anisidine | 3879.4 ± 333.9 | 2211.4 ± 467.2 | 199.1 ± 50.6 |
|  |  | veratryl alcohol | 2332.6 ± 274.7 | 2777.5 ± 560.0 | 4607.5 ± 477.8 |
| *Trametes pubescens* | BEOFB 330 | control | 1050.8 ± 264.4 | 545.3 ± 64.9 | 142.2 ± 30.1 |
|  |  | *p*-anisidine | 1738.1 ± 436.6 | 568.0 ± 50.5 | 68.3 ± 27.9 |
|  |  | veratryl alcohol | 2696.1 ± 330.7 | 5793.6 ± 345.5 | 5961 ± 605.9 |
| *Trametes suaveolens* | HAI 300 | control | 2417.8 ± 102.9 | 255.6 ± 64.5 | 162.1 ± 62.7 |
|  |  | *p*-anisidine | 1276.1 ± 197.5 | 115.5 ± 58.7 | 0.0 ± 0.0 |
|  |  | veratryl alcohol | 2694.2 ± 178.7 | 7463.5 ± 491.9 | 10438.1 ± 1604.0 |
| *Trametes versicolor* | BEOFB 320 | control | 975.1 ± 69.0 | 0.0 ± 0.0 | 108.1 ± 24.8 |
|  |  | *p*-anisidine | 1060.3 ± 125.5 | 51.1 ± 13.9 | 341.3 ± 1.0 |
|  |  | veratryl alcohol | 2431.0 ± 646.4 | 2607.1 ± 293.5 | 9667.3 ± 76.6 |
|  | BEOFB 321 | control | 2893.0 ± 313.1 | 496.1 ± 78.2 | 716.7 ± 121.1 |
|  |  | *p*-anisidine | 1255.3 ± 269.5 | 96.6 ± 23.2 | 1456.2 ± 167.6 |
|  |  | veratryl alcohol | 1721.0 ± 129.9 | 5199.1 ± 527.4 | 16291.4 ± 405.9 |
|  | BEOFB 322 | control | 1473.0 ± 430.9 | 769.6 ± 201.7 | 0.0 ± 0.0 |
|  |  | *p*-anisidine | 2207.6 ± 492.1 | 0.0 ± 0.0 | 0.0 ± 0.0 |
|  |  | veratryl alcohol | 1955.8 ± 204.3 | 2540.9 ± 272.9 | 5096.7 ± 339.8 |

**Supplementary Table S2.** Capacity of lignin, hemicellulose and cellulose degradation depending on tested *Trametes* spp. strains and treatment with inducers. Data represent mean ± S.E., n=5.

| Species | Strain | Cultivation conditions | Rate of degradation [%] | | |
| --- | --- | --- | --- | --- | --- |
|  |  |  | Lignin | Hemicellulse | Cellulose |
| *Trametes gibbosa* | BEOFB 310 | control | 48.3 ± 0.8 | 54.9 ± 0.5 | 27.1 ± 0.2 |
|  |  | *p*-anisidine | 52.0 ± 0.6 | 42.9 ± 0.5 | 31.3 ± 0.8 |
|  |  | veratryl alcohol | 32.4 ± 0.8 | 35.2 ± 0.1 | 32.0 ± 0.1 |
|  | BEOFB 311 | control | 51.8 ± 0.4 | 49.9 ± 0.8 | 36.6 ± 0.4 |
|  |  | *p*-anisidine | 48.3 ± 0.4 | 50.5 ± 0.5 | 33.9 ± 0.3 |
|  |  | veratryl alcohol | 42.3 ± 0.5 | 38.4 ± 0.4 | 40.4 ± 0.5 |
| *Trametes hirsuta* | BEOFB 30 | control | 39.6 ± 0.5 | 46.6 ± 0.2 | 24.2 ± 0.6 |
|  |  | *p*-anisidine | 56.0 ± 0.2 | 42.9 ± 0.2 | 17.4 ± 0.4 |
|  |  | veratryl alcohol | 48.9 ± 0.6 | 44.9 ± 0.2 | 32.6 ± 0.1 |
|  | BEOFB 301 | control | 37.6 ± 0.8 | 43.4 ± 0.4 | 26.5 ± 0.4 |
|  |  | *p*-anisidine | 41.6 ± 0.5 | 49.3 ± 0.9 | 14.7 ± 0.3 |
|  |  | veratryl alcohol | 43.6 ± 0.7 | 48.3 ± 0.1 | 39.9 ± 0.4 |
| *Trametes multicolor* | HAI 426 | control | 37.2 ± 0.7 | 49.2 ± 0.6 | 32.1 ± 0.4 |
|  |  | *p*-anisidine | 35.6 ± 1.2 | 37.6 ± 0.7 | 38.1 ± 1.2 |
|  |  | veratryl alcohol | 35.6 ± 0.6 | 37.6 ± 0.2 | 38.1 ± 0.3 |
|  | HAI 540 | control | 31.3 ± 0.3 | 47.3 ± 0.6 | 40.2 ± 0.4 |
|  |  | *p*-anisidine | 36.6 ± 0.6 | 39.7 ± 0.5 | 42.7 ± 0.3 |
|  |  | veratryl alcohol | 36.6 ± 0.5 | 39.7 ± 0.5 | 38.8 ± 0.3 |
| *Trametes pubescens* | BEOFB 330 | control | 44.1 ± 0.8 | 45.8 ± 0.3 | 17.2 ± 0.4 |
|  |  | *p*-anisidine | 40.2 ± 0.7 | 37.4 ± 0.5 | 20.5 ± 0.6 |
|  |  | veratryl alcohol | 40.2 ± 0.5 | 37.4 ± 0.3 | 20.5 ± 0.3 |
| *Trametes suaveolens* | HAI 300 | control | 43.9 ± 0.5 | 38.3 ± 1.0 | 24.4 ± 0.6 |
|  |  | *p*-anisidine | 33.0 ± 0.9 | 32.0 ± 0.6 | 31.3 ± 0.4 |
|  |  | veratryl alcohol | 36.4 ± 0.7 | 41.5 ± 0.8 | 37.2 ± 0.4 |
| *Trametes versicolor* | BEOFB 320 | control | 41.9 ± 1.2 | 49.1 ± 0.8 | 29.4 ± 0.6 |
|  |  | *p*-anisidine | 38.5 ± 0.2 | 41.7 ± 0.4 | 36.2 ± 0.4 |
|  |  | veratryl alcohol | 41.0 ± 0.6 | 43.0 ± 0.4 | 43.3 ± 0.4 |
|  | BEOFB 321 | control | 45.4 ± 0.3 | 39.5 ± 0.8 | 23.3 ± 0.2 |
|  |  | *p*-anisidine | 43.9 ± 0.4 | 46.9 ± 0.4 | 33.5 ± 0.2 |
|  |  | veratryl alcohol | 41.2 ± 0.6 | 43.2 ± 0.3 | 36.7 ± 0.2 |
|  | BEOFB 322 | control | 46.7 ± 0.5 | 51.6 ± 0.3 | 34.6 ± 0.3 |
|  |  | *p*-anisidine | 50.0 ± 0.8 | 44.8 ± 0.3 | 24.2 ± 0.4 |
|  |  | veratryl alcohol | 52.4 ± 0.5 | 53.4 ± 0.3 | 47.0 ± 0.5 |
